# Breathing Patterns and Oxygenation Saturation During Sleep in Children Habitually Living at High Altitude in the Andes: A Systematic Review

**DOI:** 10.3389/fped.2021.798310

**Published:** 2022-02-28

**Authors:** Santiago Ucrós, Javier A. Castro-Guevara, Catherine M. Hill, Jose A. Castro-Rodriguez

**Affiliations:** ^1^Department of Pediatrics, School of Medicine, Universidad de los Andes, Fundación Santa Fe de Bogotá, Bogotá, Colombia; ^2^School of Medicine, Pontificia Universidad Católica de Chile, Santiago, Chile; ^3^School of Clinical and Experimental Sciences, University of Southampton, Hampshire, United Kingdom; ^4^Department of Pediatric Pulmonology, School of Medicine, Pontificia Universidad Católica de Chile, Santiago, Chile

**Keywords:** sleep, high altitude, apnea—hypopnea index, polysomnography, children

## Abstract

**Background:**

Human respiratory physiology changes significantly in high altitude settings and these changes are particularly marked during sleep. It is estimated that 170 million people live above 2,500 m in environments where normal sleep parameters differ from those established at sea level or low altitude.

**Methods:**

We conducted a systematic review of publications reporting sleep studies in healthy children living at high altitude. For this purpose, data from PubMed, EMBASE, SciELO and Epistemomikos bases were retrieved up to August 2021.

**Results:**

Six articles met specified inclusion criteria; all reporting data were from South America involving 245 children (404 sleep studies) in children aged 0.6 months to 18 years, at altitudes between 2,560 to 3,775 m. The main results were: (1) Central apnea index decreased as the age increased. (2) The obstructive apnea/hypopnea index showed a bimodal profile with an increase in young infants up to age of 4 months, decreasing to 15 months of age, and then a second peak in children aged 4 to 9 years of age, dropping in older schoolchildren and adolescents. (3) Periodic breathing in the first months of life is more marked with increasing altitude and decreases with age.

**Conclusions:**

There are few studies of sleep physiology in children living at high altitude. The international parameters defining normal apnea indices currently used at low altitude cannot be applied to high altitude settings. The interpretation of sleep studies in children living at high altitude is complex because there are important developmental changes across childhood and a wide range of altitude locations. More normative data are required to determine thresholds for respiratory pathology at a variety of high altitude settings.

## Introduction

In the last three decades sleep medicine has made great progress and normal sleep normal parameters for central and obstructive apneic events (both in terms of how they are defined as well as age-specific normal values) are well-established in children living at low altitude (1–3). These values, obtained through polysomnography or cardiorespiratory polygraphy (hereon termed “sleep studies”), are a basic tool for the diagnosis of sleep disordered breathing (SDB). However, these normative values cannot be applied to high altitude where sleep physiology changes (4–9). An estimated 170 million people live at high altitude that is, living 2,500 m or more above sea-level (10). In this paper we review sleep studies conducted in healthy children and adolescents at altitudes above 2,500 m in order to inform the medical evaluation and treatment of SDB in children resident at high altitude.

## Methods

We followed the Preferred Reporting Items for Systematic Reviews and Meta-Analyses (PRISMA) guidelines to perform this review (11). We identified published studies in MEDLINE, EMBASE, SciELO and EPISTEMONIKOS databases (up to August 2021), using the terms: “Sleep in children at high altitude” OR “Polysomnography in children at high altitude” OR “Polysomnography in children above sea level” OR “Respiratory sleep polygraphy in children at high altitude” OR “Respiratory sleep polygraphy in children above sea level” restricted to child (birth to 18 years old), without language restriction. Studies published solely in abstract form were excluded because the methods and results could not be fully analyzed.

To be included, studies had to meet all the following criteria: (I) cross-sectional or cohort studies; (II) inclusion of children from birth to 18 years of age) or in a mixed population (adults and children) if children were analyzed separately; (III) experimental or intervention studies where a polysomnography or polygraphy was undertaken in healthy children living above 2,500 m sea level. Exclusion criteria were studies of children with SBD, craniofacial malformations, genetic respiratory and neurological diseases. Only studies of children expected to be acclimatized were included, for this reason research related to sojourners was excluded. Reviews and letters to the editor (without data reports) were also excluded.

Data extraction and assessment of risk of bias: Titles, abstracts, and citations were independently analyzed by three authors (S.U., J.C.G, and J.C.R.). Based on the full text form, all the studies were evaluated for inclusion criteria, population included, study design, and outcomes. After obtaining full reports about potentially relevant studies, eligibility was assessed. Disagreements were discussed and resolved by consensus, and when necessary, advice was sought from the fourth reviewer (C.M.H.). A prespecified data analysis included year, location, number of participants, age, and type of sleep study (polysomnography/polygraphy). Parameters included were central apnea index (CAI), obstructive apnea/hypopnea index (OAHI), oxygen desaturation index (ODI) either of 3% or 4%, periodic breathing % (PB), microarousal index and CO_2_ values, with their central and dispersion values. Additionally, we compared our data with sleep parameters from sea level or low altitude studies (1, 2, 12, 13).

## Results

**Figure 1:**
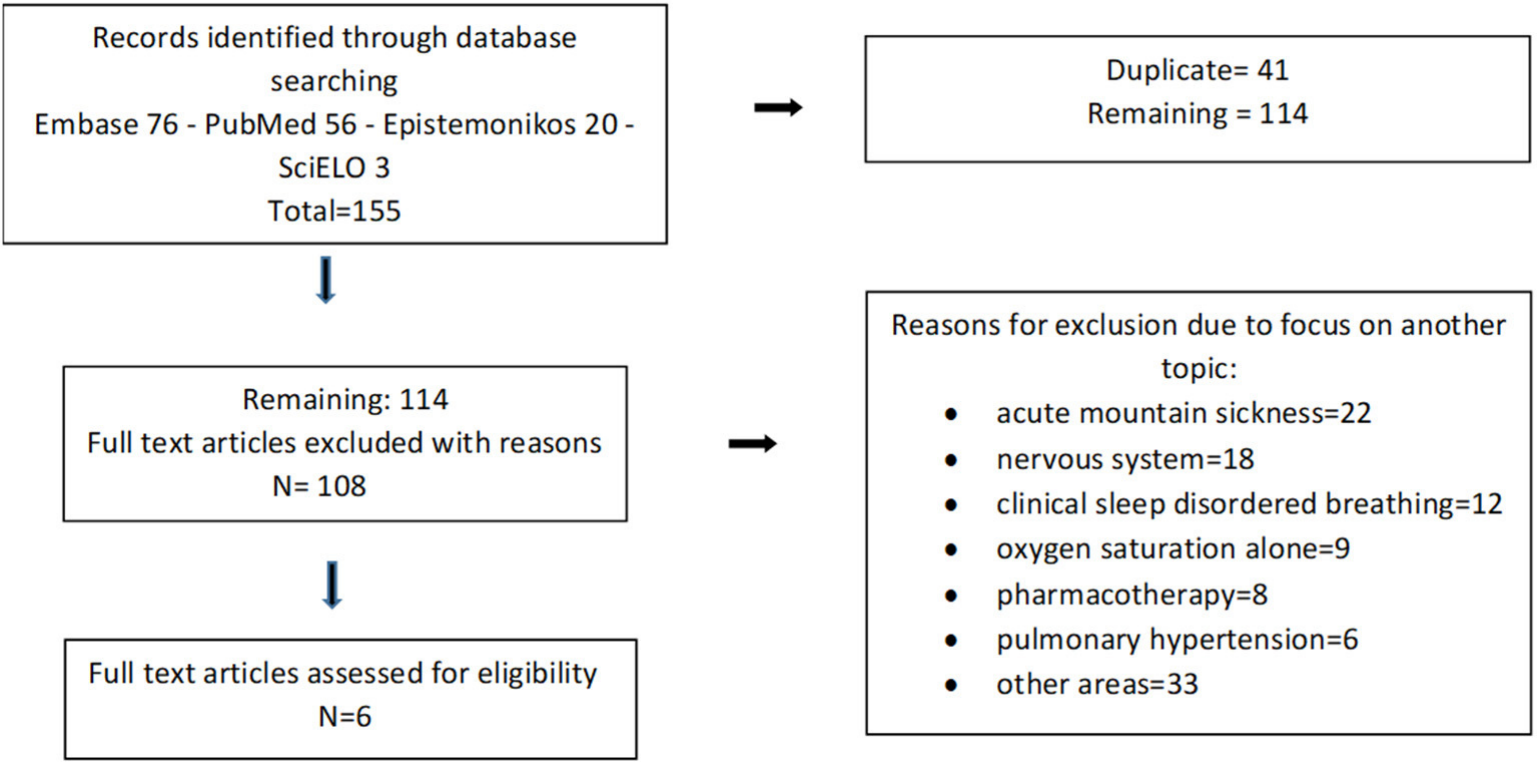
Process of study selection.

One hundred and forty-four studies were retrieved from the databases, of which 6 were eligible for inclusion (Figure 1). All articles included were from research conducted in Andean regions of South America: two in Colombia, two in Ecuador, one in Argentina and one in Bolivia (4–9). The age range was 0.6 months to 16 years, and altitudes were between 2,560 to 3,775 m above sea level. A total of 245 children underwent 404 sleep studies (Table 1).

**Table 1:** Sleep studies in children living at high altitude.

**References**	**Settlement**	**Altitude**	***n***	**Age**	**Study design**	**Type of sleep study**	**Scoring criteria**
Alducín et al. (4)	San Antonio de Los Cobres—Argentina	3,775 m	12	0.6–7.7 months	Prospective cross sectional	Polysomnography	AASM, 1992
Ucrós et al. (5)	Cuenca—Ecuador	2,560 m	35	1–4 months	Prospective cross sectional	Polysomnography	AASM, 2012
Dueñas-Meza et al. (6)	Bogotá—Colombia	2,640 m	122^*^	0.7–15 months	Prospective cohort	Polysomnography	AASM, 2012
Hill et al. (7)	La Paz—Bolivia	3,650 m	26	7–10 years & 13–16 years	Prospective cross sectional	Polysomnography	AASM, 2007
Ucrós et al. (8)	Cañar—Ecuador	3,200 m	18	1–4 months	Prospective cross sectional	Polysomnography	AASM, 2012
Ucrós et al. (9)	Chiquinquirá—Colombia	2,560 m	32	4–9 years	Prospective cross sectional	Polygraphy	AASM, 2012

*Altitude given in meters above sea level*.

* *Involved 281 sleep studies. AASM, American Academy of Sleep Medicine*.

In Tables 2–4 normal sleep parameters are presented according to age and altitude. During the first months of age the CAI increased as the altitude was higher and decreased with age, delivering the same normative values from the sea level in children 4 to 9 years old at 2,560 m, and in those 7 to 13 years old, at 3,650 m (Table 2). The OAHI showed a bimodal profile with an increase in young infants, in comparison with low altitude, a further decrease in infants up to 15 months, a new rise in children 4 to 9 years of age, and again a drop in older scholars and adolescents (Table 3). PB was only seen in the first 4 months of age, and it increased with increasing altitude (Table 4).

**Table 2:** Central apnea index (CAI) according to altitude and age.

**References**	**Settlement**	**Altitude**	**Age**	**CAI/h—Median—Dispersion**
Ucrós et al. (5)	Cuenca Ecuador	2,560 m	1–4 months	23.7 (p5 0.9–p95 130.2)
Ucrós et al. (9)	Chiquinquirá—Colombia	2,560 m	4–9 years	0.4 (p5 0–p95 2.4)
Dueñas-Meza et al. (6)	Bogotá—Colombia	2,640 m	1.0 ± 0.3 months	12.4 (p5 2.2–p95 65.4)
Dueñas-Meza et al. (6)	Bogotá—Colombia	2,640 m	3.6 ± 0.5 months	8.3 (p5 1.6–p95 50.7)
Dueñas-Meza et al. (6)	Bogotá—Colombia	2,640 m	6.6 ± 0.6 months	5.5 (p5 0.8–p95 17.8)
Dueñas-Meza et al. (6)	Bogotá—Colombia	2,640 m	13.2 ± 1.9 months	2.3 (p5 0.7–p95 8.7)
Ucrós et al. (8)	Cañar—Ecuador	3,200 m	1–4 months	30.5 (p5 8.8–p95 217.5)
Hill et al. (7)	La Paz—Bolivia	3,650 m	7–10 y 13–16 years	0.7 (IQR)

*Altitude given in meters above sea level. p, percentile; IQR, interquartile range*.

**Table 3:** Obstructive apnea/hypopnea index (OAHI) according to altitude and age.

**References**	**Settlement**	**Altitude**	**Age**	**OAHI Median—Dispersion**
Ucrós et al. (9)	Chiquinquirá—Colombia	2,560 m	4–9 years	8.8 (p5 1.21–p95 21.2)
Dueñas-Meza et al. (6)	Bogotá—Colombia	2,640 m	1.0 ± 0.3 months	6.8 (p5 0.6–p95 27.6)
Dueñas-Meza et al. (6)	Bogotá—Colombia	2,640 m	3.6 ± 0.5 months	3.5 (p5 0.3–p95 15.1)
Dueñas-Meza et al. (6)	Bogotá—Colombia	2,640 m	6.6 ± 0.6 months	0.9 (p5 0.0–p95 4.9)
Dueñas-Meza et al. (6)	Bogotá—Colombia	2,640 m	13.2 ± 1.9 months	0.5 (p5 0.0–p95 1.8)
Hill et al. (7)	La Paz—Bolivia	3,650 m	7–10 years & 13–16 years	2.1 (IQR 3.5)

*Altitude given in meters above sea level. OAHI, Obstructive apnea/hypopnea index; p, percentile; IQR, interquartile range*.

**Table 4:** Periodic breathing (PB) values according to altitude and age.

**References**	**Settlement**	**Altitude**	**Age**	**PB Median–Dispersion**
Ucrós et al. (5)	Cuenca—Ecuador	2,560 m	1–4 months	4.9% (p5 0.2%–p95 46.8%)
Ucrós et al. (8)	Cañar—Ecuador	3,200 m	1–4 months	7.2% (p5 1.2%–p95 78.7%)
Dueñas-Meza et al. (6)	Bogotá—Colombia	2,640 m	1.0 ± 0.3 months	2.0% (p5 0%–p 95 21.9%)
Dueñas-Meza et al. (6)	Bogotá—Colombia	2,640 m	3.6 1 ± 0.5 months	0.9% (p5 0%–p 95 5.7%)
Hill et al. (7)	La Paz—Bolivia	3,650 m	7–10 y 13–16 years	0% (IQR)

*Altitude given in meters above sea level. p, percentile 95; IQR, interquartile range*.

Oxygen desaturation index was higher in children 4–9 years old resident at high altitude at 2,560 m compared to similar age children at low altitude. In children 7–10 years of age living at 3,700 m a high ODI was also found (see Supplementary Table 2). With respect to CO_2_, one study reported transcutaneous measures, with a median value of 39.4 in 32 children from 4 to 9 years of age at 2,560 m of altitude (see Supplementary Table 2). Two studies reported microarousals; at 2,640 m microarousals had a median of 19/h in infants 1.0 ± 0.3 months of age with a decrease to 9.5/h in those 13.2 ± 1.9 months old (6); at 3,650 m a median of 5.5 was found in a group of 26 children 7–10 and 13–16 years of age.

## Discussion

In this systematic review, we report sleep studies in healthy children resident at various high-altitude locations in the South American Andean region. Important differences were found in all indices compared to published normative values at low altitude, except for transcutaneous CO_2_ values. As follows we discuss these findings and their implications.

Central apnea index was apparently increased in infants up to 4 months of age at 2,560 m, 2,640 m, and 3,200 m. Nevertheless, when the events associated with PB were discounted, the CAI values from sea level and high altitude were similar (see Supplementary Table 1); although at 3,650 m, where PB was not seen in schoolchildren and adolescents, a trend toward more central apnea was found (7). In relation to OAHI, values were higher than normative values in infants up to 6 months of age at 2,640 m (6), in children 4 to 9 years old at 2,560 m (9), and in older children and adolescents at 3,650 m (7). It has been proposed that this increase does not reflect genuine underlying airway obstruction, rather a lower threshold for categorization of hypopnoeic events that are scored when associated with oxygen desaturation. As children at high altitude have lower baseline SpO_2_ values, smaller fluctuations in underlying respiratory physiology results in larger dips leading to more events reaching scoring criteria (9). This is further supported by our findings of an increase in ODI at high altitude in comparison with low altitude in all the three studies which reported this value, increase that has even been reported at 1,600 m (12) (see Supplementary Table 2).

The rise in OAHI and ODI are particularly important in the age when adenoids and tonsils growth can be associated with obstructive apnea/hypopnea. Treatment thresholds for adenotonsillectomy are based on a combination of clinical findings and sleep study results. In children at high altitude the latter may be misleading if based on low altitude normative data. Normal OAHI and ODI values for age, and altitude where the child lives, should be used; otherwise, children with normal parameters will receive inappropriate treatment with all the inherent health and economic implications.

Only one study measured CO_2_ using transcutaneous measures. Values were only slightly lower than those seen at 1,600 m (12) and at low altitude level (13) (see Supplementary Table 2). On the other hand, the CO_2_ median (39.4 mm/Hg) was significantly higher than the value around 33.0 mm/Hg seen in blood gases in healthy adults at 2,640 m (14). This difference could be explained by different measurement techniques and the fact that the adult data were measured when awake while children were sampled during sleep. Further data is needed to determine normative values at high altitude, with calibration of transcutaneous or end-tidal measures with arterial values. This is particularly important to guide treatment in children monitored in high altitude pediatric intensive care settings.

Infants at high altitude experienced more PB than typically reported at sea level. During sleep at high altitude hypoxia may induce PCO_2_ drops below the apneic threshold (15). PB is seen at low altitude in healthy infants in the first months of life and disappears after 4 to 6 months of age, both at low (3) and high altitude at least up to 2,640 m (6). This is probably related to central nervous system (CNS) maturation (3). However, in young infants living at high altitude, the percentage of PB is higher in comparison with low altitude (5, 6, 8). It has been suggested that some individuals are prone to PB because CNS oxygen receptors have increased chemosensivity to hypoxia (15). Although some authors classified high altitude PB as a disease (15, 16), there is no evidence that PB *per se* is a pathological condition in infants (3). It is also important to underline that high altitude PB should not has to be confused with idiopathic central sleep apnea, an extremely rare disorder (15). Information about microarousals during sleep in children living at high altitude is particularly scarce. What can be said is that the current international guidelines do not apply at 2,640 m, for young infants who had a median microarousal index of 19/h.

Regarding the issue of attention and neurocognition it is recognized that intermittent hypoxia is a key pathway by which obstructive sleep apnea/ hypopnea can cause impairment (17–20). Given the fact that SpO_2_ decreases as altitude increases, and that the gap is wider during sleep (21), it would be expected this impairment would be a rule in children living at significant hypoxic environments. Nevertheless, the literature does not support this statement, as demonstrated by Virués-Ortega et al. (22) who reported in 41 children aged 6 to 16 years that attention skills did not differ significantly between low altitude and 3,700 m; indeed, only subtle neurocognitive differences impacts were seen. Only at higher altitudes of 4,100 m a negative outcome was found related with executive functions in a group of 8 children and 13 adolescents (22). This is perhaps surprising as the neurocognitive effects of SDB would be predicted to be more marked at high altitude due to the combination of an intermittent hypoxia in addition to basal hypoxia (7, 21, 23). The preliminary data infer a protective or adaptive mechanism in these high-altitude child populations (24).

Finally, it is important to underline that the interpretation of sleep studies at high altitude is complex because there are important developmental changes in sleep physiology and among the different ranges of altitude. Importantly however, the practice of applying normative data to interpret sleep studies based on American Academy of Sleep Medicine (25) and European Sleep Medicine guidelines (26) is flawed and such an approach would cause a significant proportion of healthy children to be erroneously diagnosed with SDB.

### Limitations of This Review

This review was limited by the relative paucity of studies describing normal sleep parameters in children living at high altitude and by small sample sizes for most included studies. Secondly all the publications found were conducted in the Andes mountains in South America. This issue is important because evolutionary adaptation of the human being to high altitude differs in Asia in comparison with South America (27). The findings of this systematic review should not be extrapolated to regions different from South America but likely strengthens the findings for the Andean region. Third, one study used polygraphy instead of polysomnography (9), in consequence in this research a proportion of hypopnea events could have been missed leading to under-estimation of obstructive apnea/hypopnea indices.

## Conclusions

There are few studies of sleep physiology in children living at high altitude. The parameters currently used at low altitude cannot be applied to high altitude. The interpretation of sleep studies in children living at high altitude is complex because there are important changes as across the different groups of age as well as across the ranges of altitude. Further large-scale populations studies are warranted at multiple altitude locations to confidently define normal parameters.

## Data Availability Statement

The original contributions presented in the study are included in the article/Supplementary Material, further inquiries can be directed to the corresponding author/s.

## Author Contributions

SU and JC-R contributed to the study concept, literature search, data collection, and manuscript writing. JC-G contributed to the literature search. CH contributed to the literature search, review the manuscript, and incorporated significant thought. All authors contributed to the article and approved the submitted version.

## Conflict of Interest

The authors declare that the research was conducted in the absence of any commercial or financial relationships that could be construed as a potential conflict of interest.

## Publisher's Note

All claims expressed in this article are solely those of the authors and do not necessarily represent those of their affiliated organizations, or those of the publisher, the editors and the reviewers. Any product that may be evaluated in this article, or claim that may be made by its manufacturer, is not guaranteed or endorsed by the publisher.
