# Supplementary material for: Breathing Patterns and Oxygenation Saturation During Sleep in Children Habitually Living at High Altitude in the Andes: A Systematic Review
Source: Front Pediatr. 2022 Feb 28;9:798310. doi: 10.3389/fped.2021.798310 (PMC8918657; doi:10.3389/fped.2021.798310)
Supplement: Supplementary file 1 [file Data_Sheet_1.pdf]

Supplementary Table 1. Central apnea index (CAI) at high altitude after discounting events related with periodic breathing and comparison with CAI values from sea level

Author/Year	Settlement	Altitude	Age	n	CAI/hour
Schlüter/2001[1]	Wittenberg Germany	Sea level	1-4 months	149 (31-60 days)<br>111 (61-90 days)<br>73 (91-120 days)	5-10
Ucrós/2015[5]	Cuenca Ecuador	2,560 m	1-4 months	35	4.5
Ucrós/2017[8]	Cañar Ecuador	3,200 m	1-4 months	18	5.4

Altitude given in meters above sea level; CAI= central apnea index

Supplementary Table 2. Oxygen desaturation index (ODI) and CO_2 in healthy children 3 to 9 years old at low and high altitude.

Author/Year	Settlement	Altitude	Age (years)	n	ODI ≥3%/hour	ODI ≥4%/hour	CO_2 (mm Hg)
Montgomery-Downs 2006[13]	Louisville USA	142	3-5	173	NA	NA	NA
Montgomery-Downs 2006[13]	Louisville USA	142	6-7	362	NA	NA	NA
Montgomery-Downs 2006[13]	Louisville USA	142	3-7	535	NA	0.4 ± 0.78	40.7 ± 4.5
Scholle[2]	Apolda Germany	6 - 447	4.3- 5.8	25	0.1	NA	NA
Burg 2013[12]	Denver USA	1,600	3-5	18	8.0	4.0 [1.4 - 0 7.7]¶	42.8 [38.3 - 46.4]¶
Ucrós 2021[9]	Chiquinquirá Colombia	2,560	4-9	32	11.2	3.9 [2.3 - 5.7]*	39.4 [31.7 - 42.3]*
Hill/2016[7]	La Paz Bolivia	3,700	7-10		8.1	NA	NA

Altitude given in meters above sea level. Age given in years. Numbers expressed as mean ± SD, or median. * [5-95 percentile] or median. ¶ [10-90 percentile]. AHI: apnea/hypopnea index. ODI: oxygen desaturation index. NA: not available
